# A co-polarization-insensitive metamaterial absorber for 5G n78 mobile devices at 3.5 GHz to reduce the specific absorption rate

**DOI:** 10.1038/s41598-022-15221-7

**Published:** 2022-07-01

**Authors:** Saif Hannan, Mohammad Tariqul Islam, Mohamed S. Soliman, Mohammad Rashed Iqbal Faruque, Norbahiah Misran, Md. Shabiul Islam

**Affiliations:** 1 Department of Electrical, Electronic and Systems Engineering, Universiti Kebangsaan Malaysia, UKM, 43600 Bangi, Selangor Malaysia; 2 Department of Electrical Engineering, College of Engineering, Taif University, P.O. Box 11099, Taif, 21944 Kingdom of Saudi Arabia; 3 Department of Electrical Engineering, Faculty of Energy Engineering, Aswan University, Aswan, 81528 Egypt; 4 Space Science Center (ANGKASA), University Kebangsaan Malaysia, UKM, 43600 Bangi, Selangor Malaysia; 5 Faculty of Engineering, Multimedia University (MMU), 63100 Cyberjaya, Selangor Malaysia; 6 Department of Electronic and Telecommunication Engineering, International Islamic University Chittagong, Chittagong, 4318 Bangladesh

**Keywords:** Biomedical engineering, Electrical and electronic engineering

## Abstract

Specific absorption rate (SAR) by next-generation 5G mobile devices has become a burning question among engineers worldwide. 5G communication devices will be famous worldwide due to high-speed data transceiving, IoT-based mass applications, etc. Many antenna systems are being proposed for such mobile devices, but SAR is found at a higher rate that requires reduced for human health. This paper presents a metamaterial absorber (MMA) for SAR reduction from 5G n78 mobile devices at 3.5 GHz. The MMA is co-polarization insensitive at all possible incident angles to ensure absorption of unnecessary EM energies obeying the Poynting theorem for energy conservation and thus ensuring smooth communication by the devices. The unit cell size of the absorber is 0.114 $\lambda$ making it design efficient for array implementation into mobile devices. This absorber has achieved a minimum of 33% reduction of SAR by applying to the 5G n78 mobile phone model, equivalent to SAR by GSM/LTE/UMTS band mobile phones and making it suitable for SAR reduction from next-generation 5G mobile devices.

## Introduction

In the last few years, 5G mobile communication and the development of relevant devices have dragged tremendous attention among researchers worldwide. Mobile devices like cellphones, laptops, tablet PC, etc., will be equipped with an antenna for communication in 5G frequencies. The microwave or millimeter-wave frequencies in these 5G devices will mainly ensure high-speed data transceiving and other benefits. Still, they will emit a comparatively large amount of EM energies to the human user^1,2^. The human body, especially the head and hand, will be exposed to strong EM waves, leading to cell damage upon using such devices for a long time^3,4^. Thus, specific absorption rate (SAR) is the associated concern with the usage of next-generation 5G mobile devices^5,6^. SAR of these 5G devices should be as low as possible so that people will be at no risk of using 5G devices in their everyday life. The antenna design for 5G mobile devices has become a recent trend, where gain, directivity, etc., are ensured, but SAR reduction is still an alarming issue.

Metamaterial absorbers (MMA) integrated (or placed parallelly) with a 5G antenna can enhance the antenna’s performance with improved gain and desired directivity. In addition, these MMAs can absorb unnecessary EM waves and thus can reduce SAR^7^. MMA is an artificially designed metal-dielectric substrate sandwiched multilayered structure that shows unusual electromagnetic (EM) response (i.e., a negative value of either permittivity or permeability or both) with unnatural refractive index along with the absorption of the incident EM waves^8–10^. The metal layers on both sides of the dielectric substrate layer act as resonator/patch and ground. Depending on the patch design and the ground dimension, such MMAs can be perfect MMA or polarization converters (co-polarization absorber)^11,12^. If an MMA can absorb the entire applied EM wave, i.e., the co-polar and cross-polar elements of applied EM waves, it can be considered a perfect MMA^13,14^. Whereas, if an MMA can only absorb co-polarized EM waves and pass the cross-polarized EM waves through it, it can be regarded as a cross-polarization converter or co-polarization absorber^15–18^. If a perfect MMA is used with a 5G antenna, it will absorb almost the entire signal coming to or from the antenna and thus will make the antenna useless for the 5G devices for communication. Therefore, co-polarization MMAs are the only solution to absorb desired EM waves and let the antenna work properly for selected performances of the 5G devices.

The antenna system in mobile devices is designed for different target frequency bands (like GSM, UMTS, LTE, 5G) and other standard services like Bluetooth and WiFi (2.4 GHz or 5 GHz)^19,20^. The individual antenna sets are placed inside mobile devices for individual target frequencies. The SAR value of GSM/UMTS/LTE antennas is already within allowed values, but 5G antennas have shown considerably high SAR values due to higher operational frequencies^4,21^. Thus MMAs can be introduced for 5G devices to reduce SAR due to 5G antennas^22^. These MMA should be co-polarization insensitive and not be a perfect absorber. Different countries have adopted different frequency allocations for 5G communications, with low, medium, and very high frequencies. In Malaysia, 700 MHz, 3.5 GHz, and 26–28 GHz frequencies are set by the Malaysian Communications and Multimedia Commission (MCMC) for next-generation 5G communications^23^. The 3.5 GHz frequency band has already become popular among mobile operators in Malaysia^24^, and thus our target 5G frequency is 3.5 GHz.

In this study, we propose a co-polarized, incident angle-insensitive MMA at 3.5 GHz to reduce the SAR of next-generation mobile devices (5G Band n78 devices) in Malaysia. The proposed MMA was designed targeting the 3.5 GHz frequency with the least possible unit cell size and has shown metamaterial properties at this frequency. Moreover, the absorber was measured practically with VNA (vector network analyzer) to ensure its performance as per simulation. In addition, a planer sleeve monopole antenna operating at 3.5 GHz n78 devices was imported from commercially available Antenna Magus™ to CST Design Environment™ software placed inside a template-based 5G mobile phone along with the proposed MMA for SAR calculation. The template-based “SAR Head Hand and Phone” model was loaded from the “component library” of CST 2021 software. Then the monopole antenna was placed inside the mobile phone to simulate SAR calculation before and after incorporating the proposed co-polarized MMA. The reduction of SAR was found at a satisfactory level by 33% for 1 g and 35.6% for 10 g SAR calculation after placing the proposed absorber parallelly with the 5G antenna inside the 5G phone.

## Metamaterial absorber design

### Frequency targeted metamaterial absorber

Frequency-targeted antennas are common, but frequency-targeted MMAs are rare to find. To design a specific frequency targeted MMA, some factors (L, R & C components) must be considered^16^. The absorber must have a resonating patch with an inductance value associated with the necessary resistance value for required S parameters (S_11_ mainly) and the corresponding capacitive value. If a solid metal ground is used for the MM structure, the patch should have a required circumferential gap from the edges of the substrate area; so that a similar LRC component can achieve that required coupling due to the ground and its circumferential effect on the patch. Thus, it can be stated that for a single resonance frequency, there should be two parallel LRC components in the absorber unit cell. In addition, there should be shunt capacitance in between the LRC components and the waveguide ports to maintain isolation at the operating frequencies other than the resonance frequency.

### Design of the MM unit cell and its optimization process

**Figure 1:**
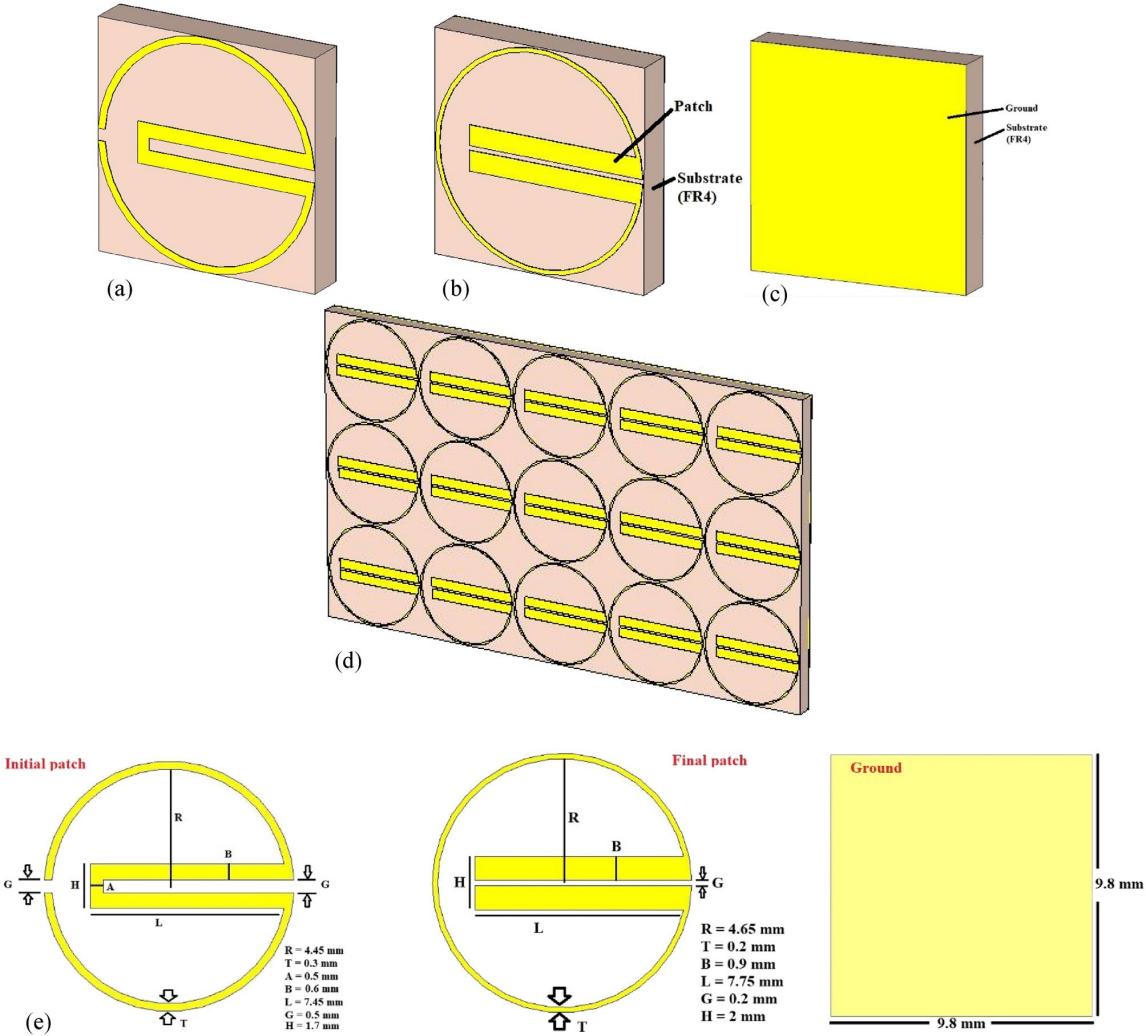
Design of the proposed metamaterial (**a**) initial patch of the unit cell (unit cell-1), (**b**) finalized patch of the unit cell (unit cell-2), (**c**) ground of the unit cell, and (**d**) array structure (3 × 5) for 5G mobile phone. (**e**) Dimension of the patch and the ground.

The unit cell aimed to have a simple circular split-ring patch structure and solid ground with the desired output. To prove the uniqueness of the patch structure, two parallel strips are added at the center with the ends of the split ring, as shown in Fig. 1a. To get a stable unit cell to replicate in the array, the unit cell was modified further by a thin circular split-ring with thick parallel strips joined at the ends of the split (Fig. 1b). The size of the unit cell is 9.8 mm × 9.8 mm, which is equivalent to $0.114 \lambda$ considering the wavelength $\lambda$ for 3.5 GHz. In an array, the matrix of unit cells can result in deviation from the desired S_11_ parameter due to mutual coupling among the neighboring unit cells. Thus, the unit cell should be designed precisely so that this mutual coupling in the array doesn’t affect the absorption and metamaterial performance.

**Figure 2:**
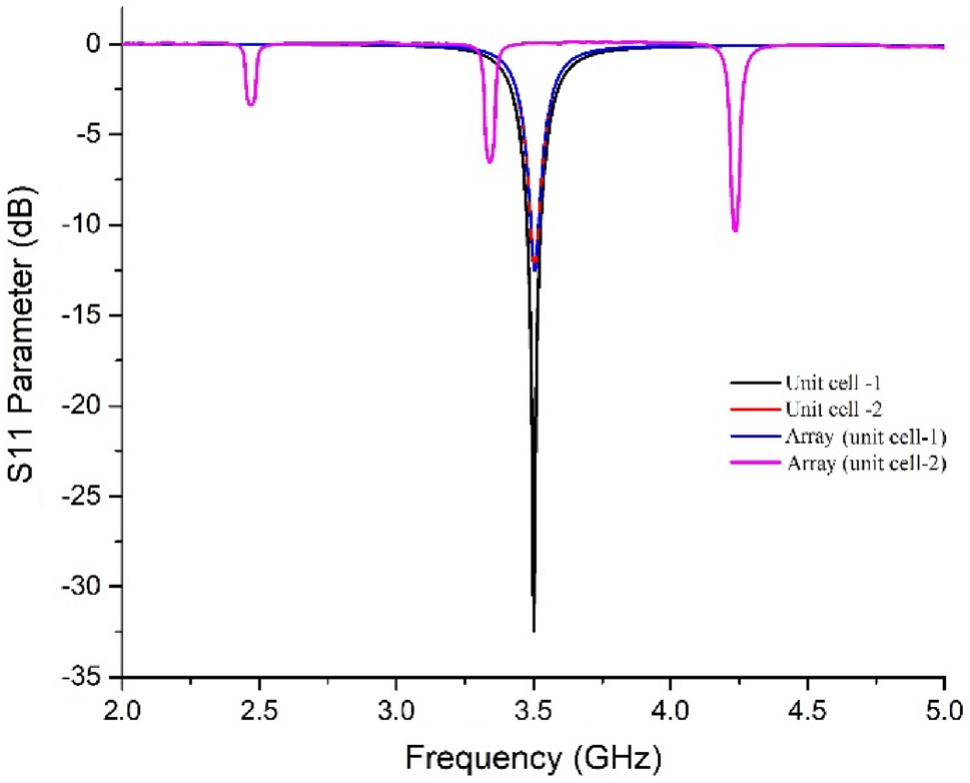
S_11_ (reflection coefficient) performance of the absorber for unit cells-1 and -2 and the corresponding array structures.

A solid ground is considered for the unit cell to ignore the transmission coefficient from the absorption equation, $A=1-{\left|{S}_{11}\right|}^{2}-{\left|{S}_{21}\right|}^{2}$ where $A$ is absorption, ${\left|{S}_{11}\right|}^{2}$ is the reflection coefficient and ${\left|{S}_{21}\right|}^{2}$ is the transmission coefficient. Both the patch and the ground are of annealed copper of thickness 0.035 mm. The substrate was chosen the commercially available FR4 (thickness 1.6 mm, relative permittivity 4.3); such a high dielectric constant helps design a metamaterial absorber with a subwavelength dimension range. The reason behind the evolution of the unit cell can be understood from the S_11_ performance of the unit cell due to the initial and final patch design and the array using the final patch (as per Fig. 1) in Fig. 2. It can be seen from Fig. 2 that the S_11_ parameter coincided at 3.5 GHz with almost similar values (in dB) for the array and the unit cell 2. Although unit cell-1 has shown the greater value of S_11_, the array using this structure has shown deviation from 3.5 GHz, and thus, the unit cell was needed to be modified to sacrifice a higher value in − dB, but at least − 10 dB values.

An array of an MMA should show the same S parameters (in this case, the S_11_ parameter) as that of the constituting unit cells (in this case, the unit cell 2) depicted in Fig. 2. Thus, it is required to know the reason for such EM behavior of the unit cell (or the array), to understand the mechanism of the proposed absorber.

### Equivalent circuit of the absorber

**Figure 3:**
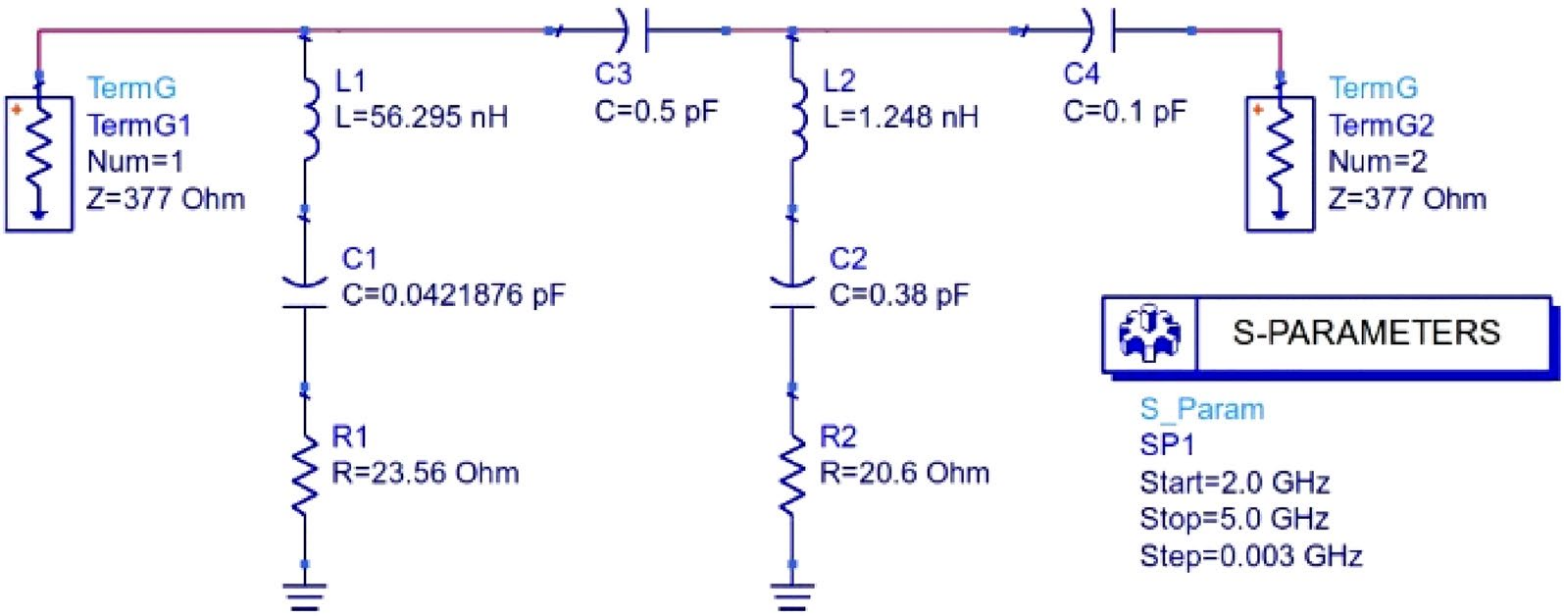
Equivalent circuit of the metamaterial absorber (unit cell/array).

The electromagnetic response of the absorber (unit cell or array) can be understood from the equivalent circuit, where the resonators are expressed as LRC circuits (Fig. 3).

**Figure 4:**
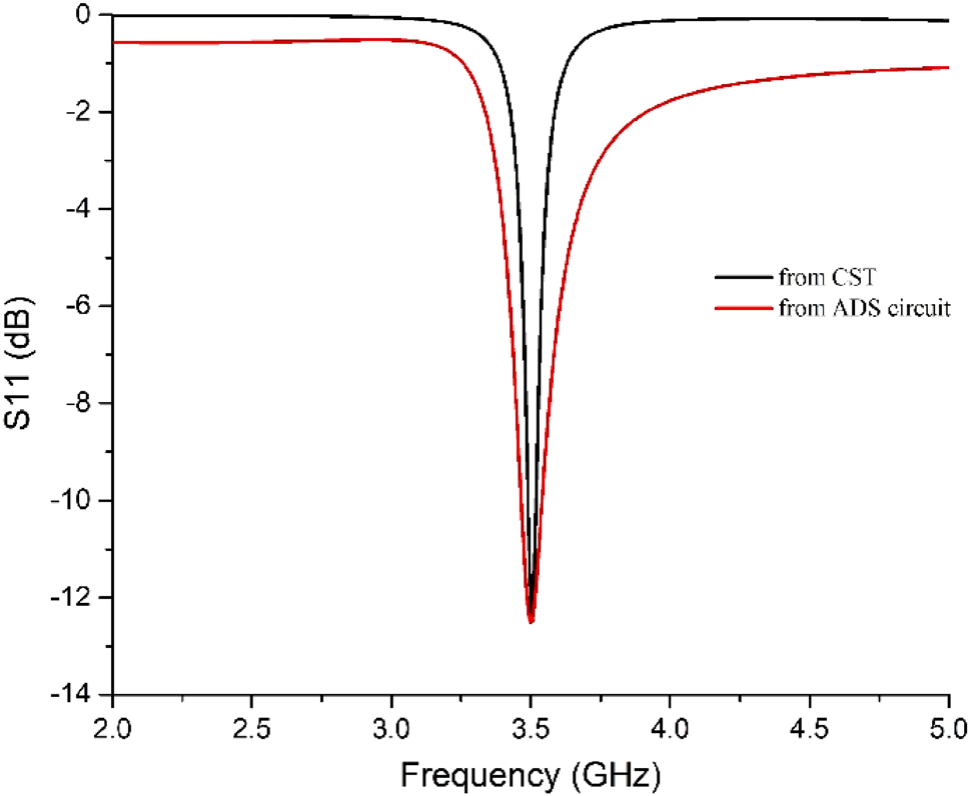
Comparison of S_11_ parameter between CST simulation and equivalent circuit.

The equivalent circuit was designed on ADS™ software. The component L1–C1–R1 corresponds to the patch shown in Fig. 1b, and L2–C2–R2 corresponds to the ground. C3 and C4 are capacitive gaps (or isolation) in between the L-R–C circuits that can be correlated with the near-zero S_11_ values (in dB) in the entire operating frequencies except for the resonance frequency (3.5 GHz) shown in Fig. 4. The waveguide ports applied at the two faces of the absorber were set at a distance of 21.44 mm from the reference plane by the CST simulator. Thus, the air gap was in between each port and the absorber face, and hence an impedance of 377 ohms was applied at each port in the equivalent circuit.

## Analysis of metamaterial characteristics at the resonance frequency

**Figure 5:**
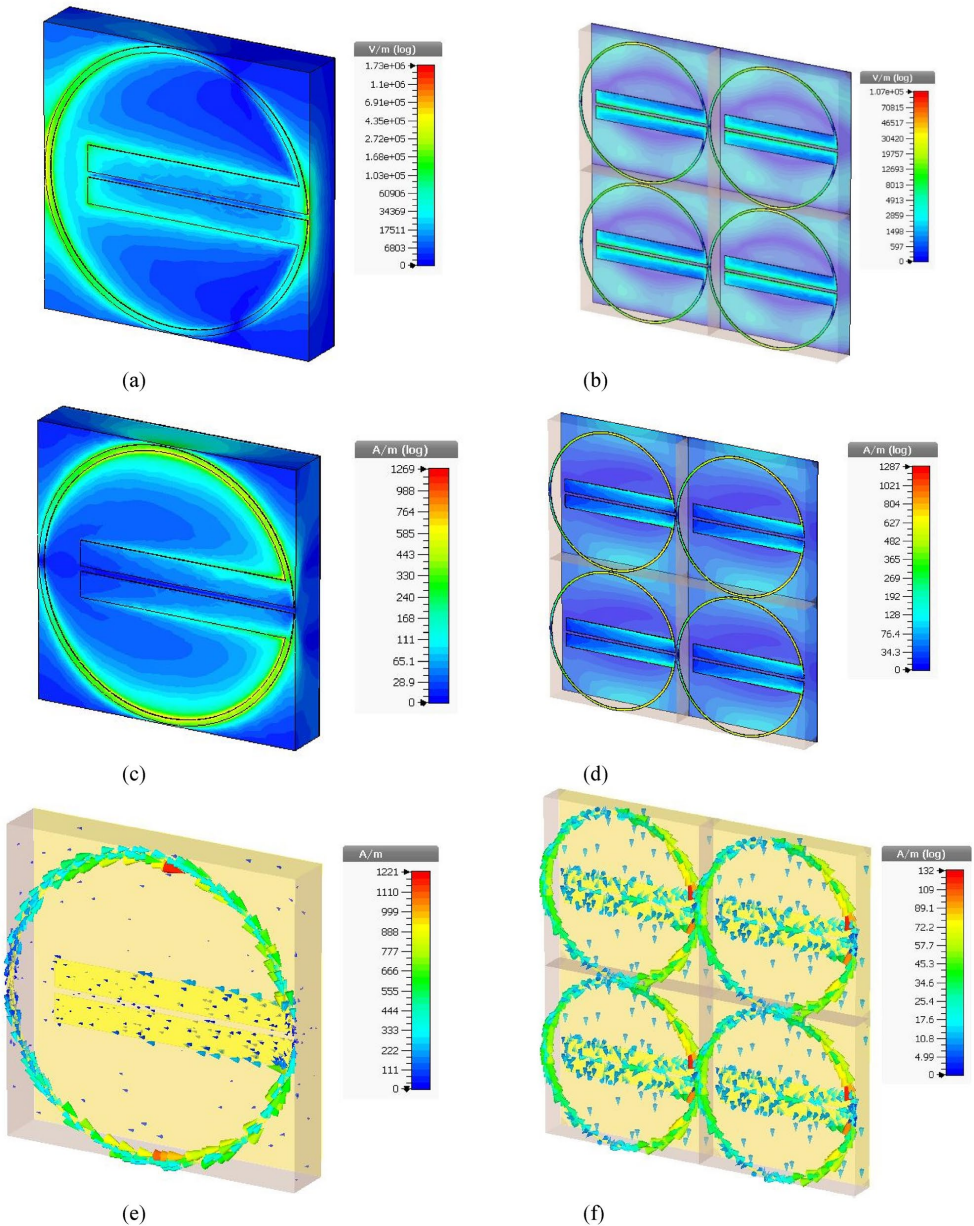
The electric field distribution of (**a**) the unit cell and (**b**) the array. The magnetic field distribution of (**c**) the unit cell and (**d**) the array. The surface current distribution of (**e**) the unit cell and (**f**) the array.

The designed absorber has shown required metamaterial properties at the resonance frequency (3.5 GHz). The resonating patch has shown visible excitation for both E-field, H-field, and surface current density in the simulation, as depicted in Fig. 5. It can be seen from Fig. 5 that the patch is agitated at the circumferential ring only, and the central parallel strips (connected at the ends of the split of the circular ring) are less agitated. It may lead to the idea that the parallel strips are useless. The central parallel strips were required to tune the resonance frequency at 3.5 GHz, i.e., to get the required inductance of the patch along with the necessary resistance and the associated capacitance for the resonance frequency. It can also be seen from Fig. 5b,d,f that there is no mutual coupling among the neighboring unit cells in the array, which was essential to maintain resonance at the desired frequency for the array structure (of any desired size), to install inside any 5G mobile devices. It is necessary to mention that the unit cell was considered along the x and the y-axis (both positive and negative directions), and open space was set along the z-axis for array simulation so that real boundary conditions are satisfied as per practical applications. The E and H fields shown in Fig. 5a,b are the E_xy_ and H_xy_ components of $\left|\mathrm{E}\right|$ and $\left|\mathrm{H}\right|$ fields.

The value of the relative permittivity and the refractive index was found negative at the resonance frequency. The absorber achieved a maximum of 94.13% absorption, as listed in Table 1, proving it a metamaterial absorber. The negative permittivity value ensures metamaterial properties with energy conservation due to the negative value of corresponding real parts.

**Table 1:** Metamaterial properties of the proposed absorber derived from simulation.

Resonance frequency	Relative permittivity	Relative permeability	Refractive index	Maximum absorption
3.5 GHz	− 2.66	1.83	− 0.01	94.37%

### Incident angle-insensitivity of the absorber

**Figure 6:**
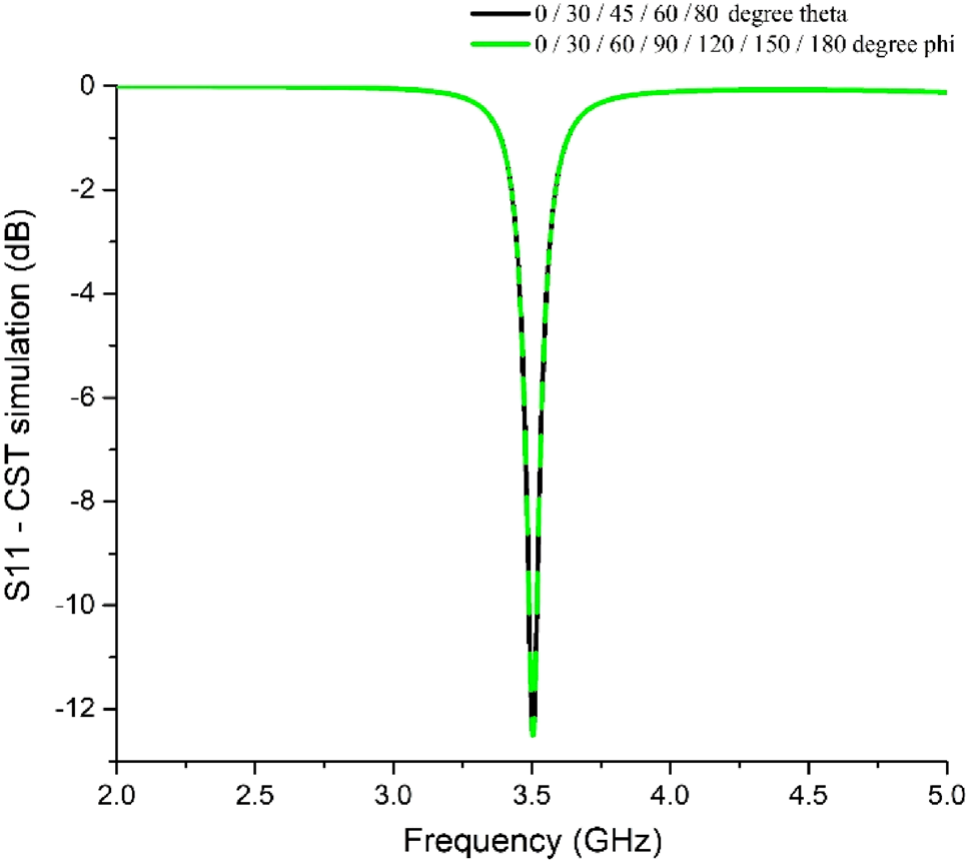
S_11_ parameter values for incident EM waves at different normal (theta polarization) and oblique (phi polarization) incidences.

The absorber should be insensitive to any angle of incidence, either at normal or oblique incidence, to effectively absorb the applied EM signal (co-polarization element only). Therefore, it is essential for the degrees of freedom of the human user with 5G mobile devices. Thus, the proposed absorber unit cell (unit cell-2) was subjected to all possible theta and phi polarization, and the results are found to be the same for the S_11_ parameter, as shown in Fig. 6. Thus the absorber is incident-angle insensitive.

## SAR simulation setup and calculation

**Figure 7:**
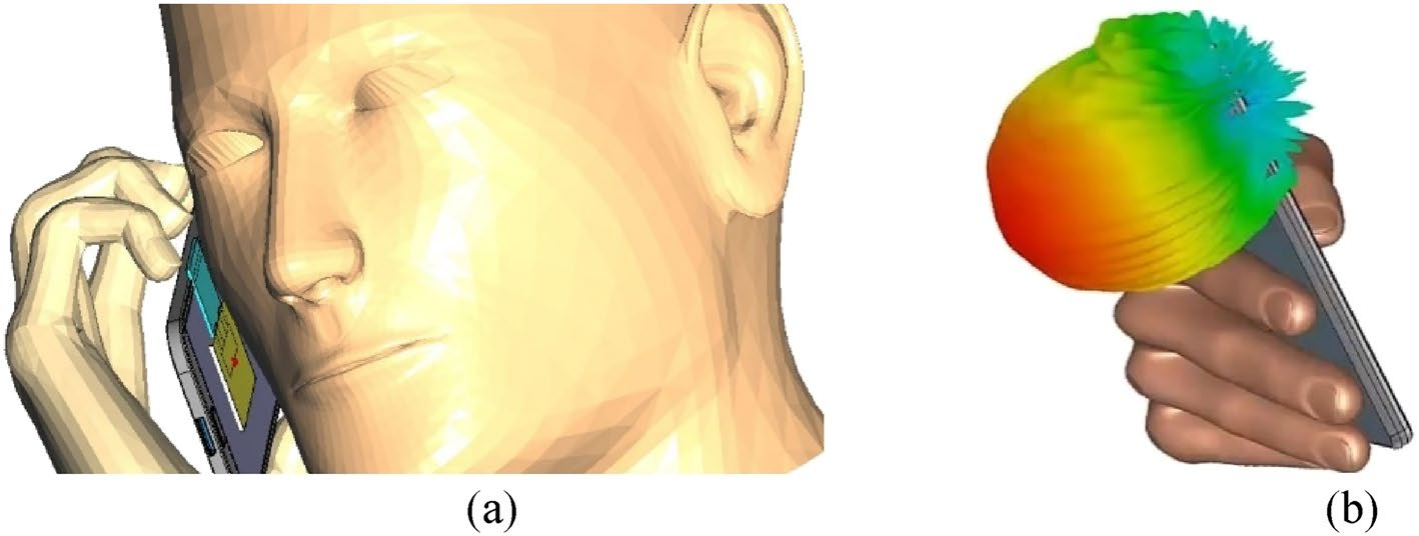
(**a**) Simulation setup of the 5G antenna and the designed MMA inside a mobile phone with human head and hand phantom (blue—antenna, yellow—MMA), (**b**) 3.5 GHz antenna radiation direction. [The images were taken from the component library of CST studio suite 2021 software and edited, URL: https://www.3ds.com/products-services/simulia/products/cst-studio-suite/].

The metamaterial was set inside a 5G n78 mobile phone placed between a human head and hand model, as shown in Fig. 7a. The metamaterial absorber was placed behind the ground of the 3.5 GHz antenna facing towards the mobile screen, i.e., towards the head phantom, to effectively absorb unnecessary EM energy emerging towards the human head. The 3.5 GHz antenna is placed inside the mobile set facing the backside, where maximum radiation will occur, as shown in Fig. 7b. The “SAR Head Hand and phone” template was loaded from the “Component Library” of CST 2021, and all the antennas in the phone were deleted. In place of an antenna slot, a template-based 5G band n78 antenna named “Planer sleeve monopole antenna” from “Antenna Magus 2021 Professional” was exported into the CST “SAR Head Hand and phone” file. The phone was simulated with this added antenna to calculate gain and SAR at 3.5 GHz without the MMA.

**Figure 8:**
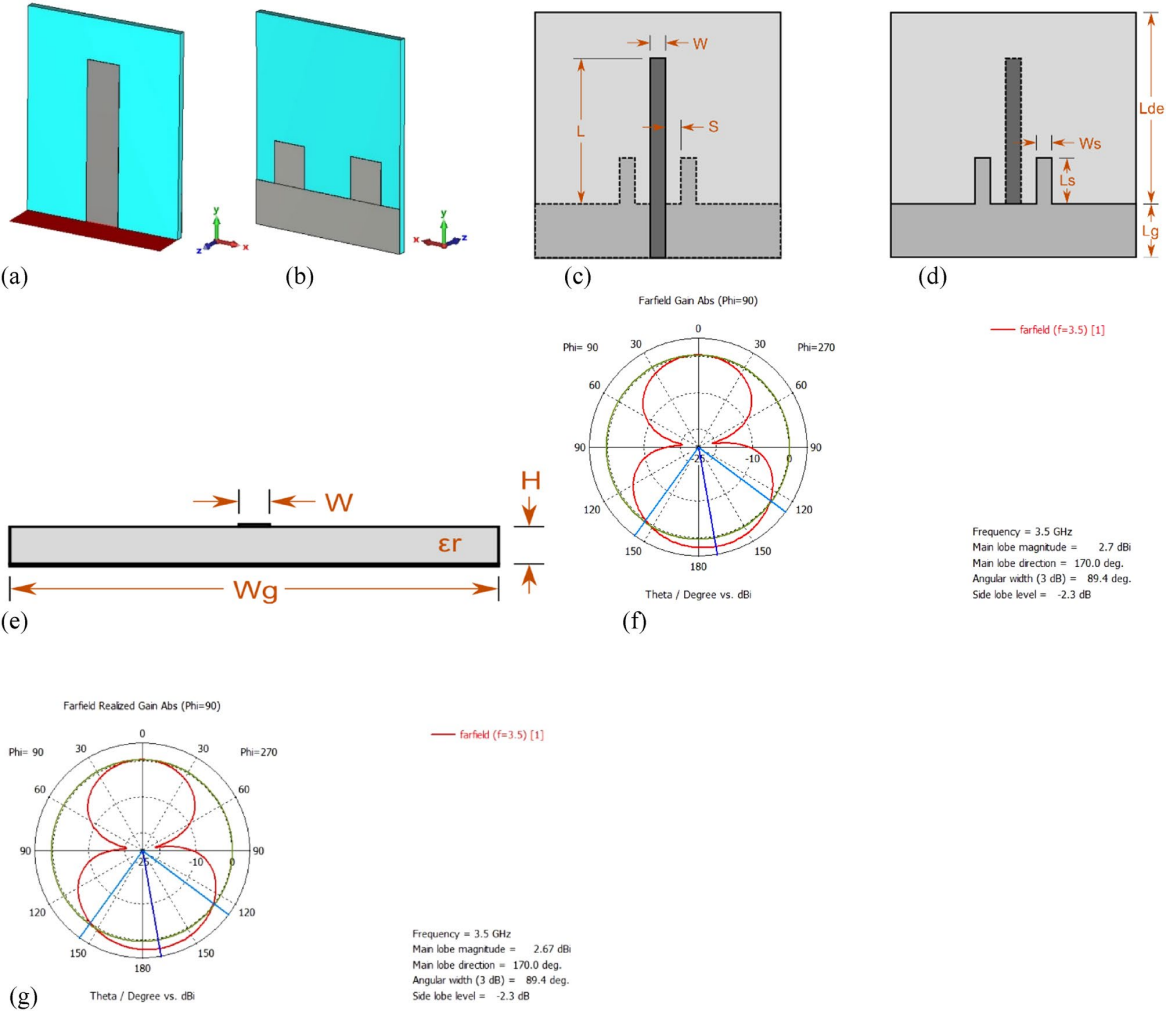
The details of the 3.5 GHz antenna (**a**) patch, (**b**) ground. Dimensions of the antenna in (**c**) top view, (**d**) bottom view, (**e**) side view, (**f**) gain, and (**g**) realized gain of the antenna.

The detailed dimension, gain, and realized gain (at 3.5 GHz) by the antenna is shown in Fig. 8. The substrate of the antenna is Rogers RT 5880. The proposed metamaterial absorber (substrate: FR4) will be placed behind the 3.5 GHz antenna without any physical attachment to the antenna. The detailed dimensions of the antenna as per Fig. 8c–e are listed in Table 2. The red-colored area shows the feeding plane of the antenna in Fig. 8a.

**Table 2:** Detailed dimension of the 3.5 GHz antenna.

W	L	Ws	Ls	S	Wg	Lg	H	Lde	${\varepsilon }_{r}$
6.192 mm	21.81 mm	6.192 mm	7.503 mm	1.639 mm	29.50 mm	7.868 mm	1.285 mm	m	2

*Length, breadth, and width of the antenna substrate are 21.86 mm, 21.81 mm, and 1.285 mm, respectively.

**Figure 9:**
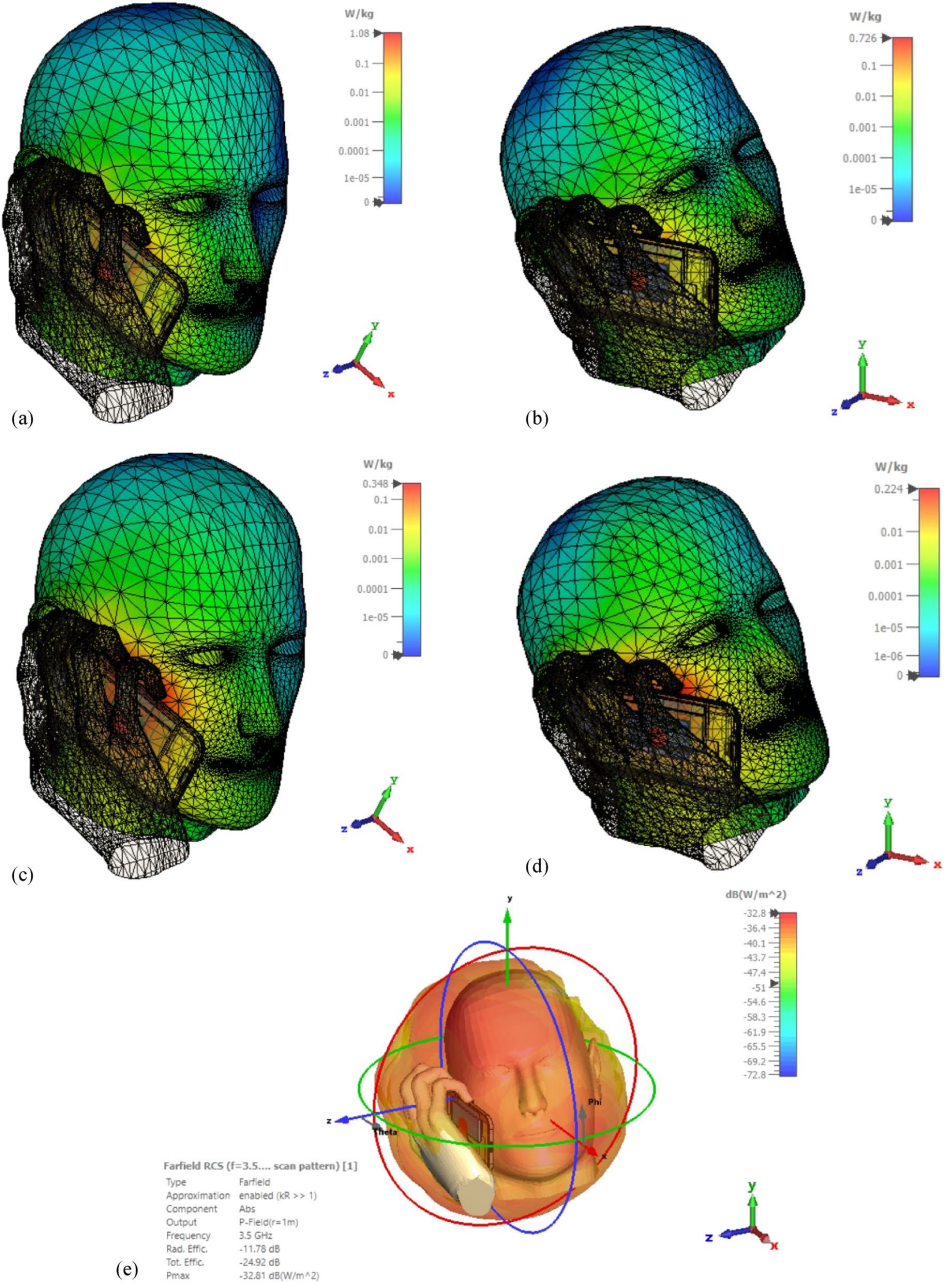
Calculated specific absorption rate (SAR) from CST simulation of the “Phantom” for 1gm averaging mass (**a**) without absorber, (**b**) with absorber, and for 10 gm averaging mass (**c**) without absorber and (**d**) with the absorber. (**e**) The maximum radiation power by the 3.5 GHz antenna in the presence of the absorber. [The images were taken from the component library of CST studio suite 2021 software and edited, URL: https://www.3ds.com/products-services/simulia/products/cst-studio-suite/].

The “SAR Head Hand and Phone” phantom was simulated for the antenna with and without the MMA. The SAR calculated by the CST simulation is shown in Fig. 9. The SAR calculation was done for the averaging mass of 10 g and 1 g of the head and hand phantom.

It can be observed from Fig. 9 that the value of SAR reduced from 1.08 to 0.726 Watts/kg considering the 10-g averaging mass for the head and hand, which is a reduction of 32.8% by the absorber. Again, the SAR was reduced from 0.348 Watts/kg to 0.224 Watts/kg, a 35.6% reduction by the absorber considering 1-g averaging mass. 0.726 Watts/kg (10 g) or 0.224 Watts/Kg (1 g) are very satisfying SAR values for high frequency-based mobile devices, comparable to SAR values of standard mobile devices using GSM, LTE, or UMTS band^25,26^. The proposed absorber was placed behind the 5G antenna facing toward the mobile screen (that faces toward the head phantom in Fig. 7), and thus it will be absorbing the backscattered EM energy only. Due to the long distance from the base station or low battery power, the antenna will radiate at maximum power but mainly towards the phone's backside. The adaptive power control option was considered by default in the simulation setup, and the SAR calculation was done accordingly. Thus the proposed absorber has shown the capability to maintain low SAR for the next generation 5G mobile devices at 3.5 GHz, specifically for n78 devices.

## Measurement of the absorber

**Figure 10:**
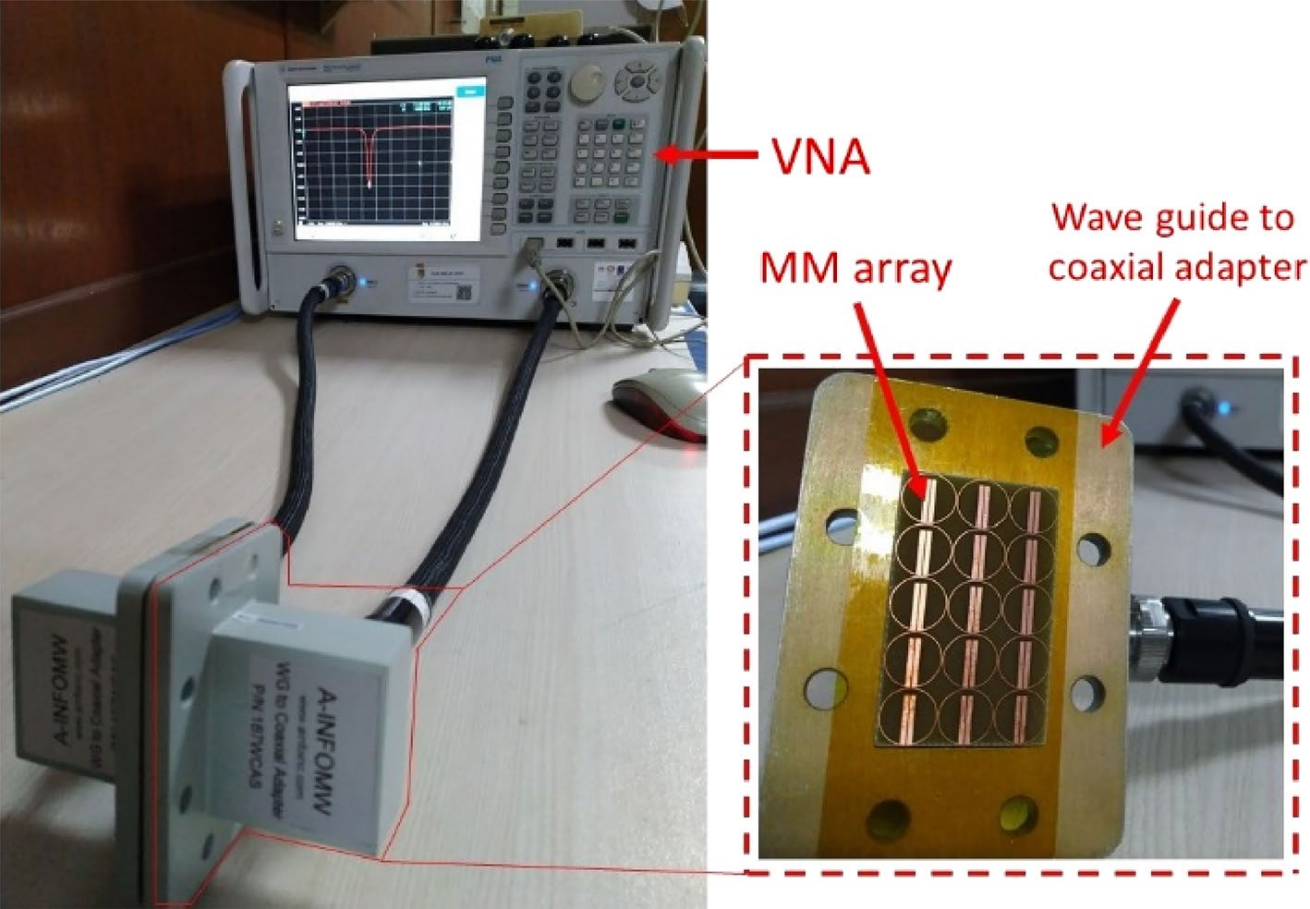
Measurement setup of the absorber prototype.

The proposed absorber was fabricated and measured to validate its S_11_ performance after successfully simulating it for absorption and SAR reduction at the resonance frequency. The array was fabricated as per the size of the aperture of a waveguide-to-coaxial adapter that was allowed for 2 to 5 GHz operation by VNA (vector network analyzer). The measurement setup is shown in Fig. 10.

**Figure 11:**
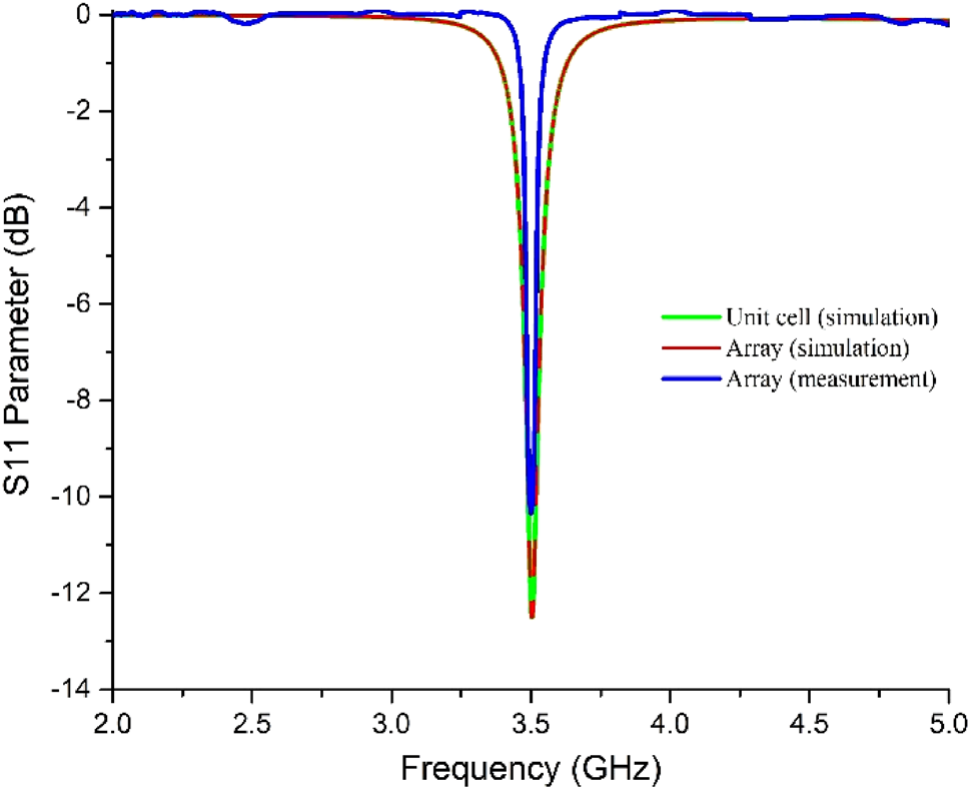
Comparison of S_11_ parameter (dB) by simulation and measurement.

It can be seen from Fig. 11 that the measured S_11_ value (in dB) fairly shows a − 10.339 dB value, whereas the simulated values were − 12.18b dB by both the unit cell (unit cell-2 in Fig. 1) and the array. This slight mismatch is acceptable as the resonance frequency is found at 3.5 GHz in both cases. Moreover, it is very normal that simulation and measured data never match perfectly due to imperfect measurement setup and loss in coaxial cables or adapters. However, there are some mismatches between the simulation and measured data at other frequencies that are negligible as the values are much less than − 10 dB. Thus the absorption by the absorber at 3.5 GHz was validated practically.

The proposed MMA has been compared with recent relevant works and found novelty in some cases as listed in Table 3. The proposed absorber was found unique in terms of both the metamaterial properties (as listed in Table 1) and maximum absorptions at 3.5 GHz which is suitable for SAR reduction from n78 5G mobile phones or other devices as discussed above. No such metamaterials are found to date that has been designed to solely target 3.5 GHz along with metamaterial properties and absorptions (as shown in Fig. 11). Some metamaterial-inspired antennas were proposed but none of them showed the effect on the resonance frequencies due to other frequency-selective absorptions by the absorber. In addition, their metamaterial properties are found at some frequencies that do not align with any defined communication frequencies. This makes the performance of these designs questionable. On the other hand, the proposed MMA does not interrupt any other frequencies of the n78 5G mobile phones as it has shown absorptions only at 3.5 GHz. Moreover, the proposed metamaterial is co-polarization insensitive (and thus non-perfect absorber) and it ensures smooth functioning of the 5G antenna due to its placement at the back of the antenna; along with desired SAR reduction from human tissues exposed by the back lobe from the antenna.

**Table 3:** Comparison of proposed MMA with relevant works for SAR reduction.

Ref.	Publication year	Design of metamaterial patch	Material and thickness of substrate	Dimension of MMA	Type of Antenna used	Verified by proper equivalent circuit	Metamaterial property with absorption at 3.5 GHz (For n78 devices)	Novelty
^27^	2021		FR4 1.574 mm	5 × 5 ${\mathrm{mm}}^{2}$	Dipole-inspired patch antenna	No	No	Metamaterial antenna for 4G, 5G, and NB-IoT applications
^28^	2021		FR4 1.574 mm	5 × 5 ${\mathrm{mm}}^{2}$	Folded dipole antenna	No	No	Metamaterial antenna for 4G and 5G applications
^22^	2021		FR4 1.6 mm	14 × 14 ${\mathrm{mm}}^{2}$	No specific antenna was proposed	No	No	Random absorptions at L, S, and C Bands
This work	2022		FR4 1.6 mm	9.8 × 9.8 ${\mathrm{mm}}^{2}$	Planer sleeve monopole antenna	Yes	Yes	Metamaterial absorber designed solely at 3.5 GHz for SAR reduction (from n78 devices)

## Conclusion

In this study, we numerically studied the SAR reduction from next-generation 5G n78 mobile devices by applying a novel co-polarization-insensitive metamaterial absorber (MMA) and experimentally verified the absorptivity of the MMA at the resonance frequency. The MMA was designed to aim the resonance frequency at 3.5 GHz with metamaterial characteristics by the necessary engineering of L-C-R transmission lines as per the equivalent circuit. However, it is essential to mention that the metamaterial was designed with a modified circular split-ring resonator so that it cannot be rotationally symmetric^13,29^ and thus be able to absorb the co-polarized portion of the applied EM wave only. This property made it appropriate to be used inside 5G mobile phones for both smooth communication and SAR reduction. However, as perfect MMA can absorb entire applied EM waves of both the co-polar and the cross-polar elements, they will not be suitable for applying to mobile phones or other similar devices due to the entire signal absorption capability that can hamper EM signal, even if they can reduce SAR effectively. Furthermore, the proposed MMA has shown at least a 33% reduction of SAR from n78 5G mobile phones and maintained the SAR value equivalent to GSM/ LTE/ UMTS bands for the 5G band, a benchmark set by the proposed absorber.

## Methods

### Simulation methodology

The absorber was simulated on CST 2021 software. “Phase reflection diagram” workflow was followed with the “frequency domain” solver for simulation. Perfect electric field was applied along the x-axis, perfect magnetic field along the y-axis, and open space (plane EM wave) along the z-axis as boundary conditions for the unit cell. The waveguide ports were set along the z-axis with a solver-generated distance from the absorber surfaces. For array simulation, “unit cell” was set along the x- and y-axis and open space (plane EM wave) along the z-axis as boundary conditions. Floquet ports were applied along the z-axis, and both Zmax and Zmin modes were considered during the simulation.

The equivalent circuit was designed and simulated on ADS 2017 software. The “Simulation-S_param” mode was chosen with a two-port terminal set-up to design the equivalent circuit. For the desired (3.5 GHz) frequency, the L–C–R values were chosen per the corresponding patch structure calculation. As per CST simulation, two capacitive gaps were set between the L–C–R lines and the ground for isolation. To match the number of data samples of CST, the simulation was set from 2 to 5 GHz with a step of 0.003 GHz. Finally, the S parameters were extracted from a template-based solver and then exported in a txt file to do a further graphical representation of the S parameters.

### Unit cell optimization for placement in the array

The unit cell was optimized from the initial to the final patch structure (shown in Fig. 1) by calculating capacitance between the patch perimeter and the ground perimeter through the substrate. Moreover, the values of inductance and capacitance of the patch resonator (values are shown in the equivalent circuit in Fig. 3) were calculated using the following Eqs. ^16^.

1 $${L}_{ms}=0.00508L \left[\mathrm{ln}\left(\frac{2l}{W+D}\right)+0.5+0.2235 \left(\frac{W+D}{l}\right)\right],$$

where ${L}_{ms}$ is the inductance per unit length of the microstrip $(\mu H)$, *l* is the length of the strip (inches), W is the width of the strip (inches), and D is the distance between the stripline and the ground plane.

2 $$C= \frac{1}{4{\pi }^{2}{f}^{2}{L}_{ms}},$$

where $f$ is the desired frequency (here 3.5 GHz).

The R values in the circuit were found from the adjustment /tuning of R on the ADS circuit simulator to get the desired value of the S_11_ parameter.

The capacitance between the patch perimeter and ground perimeter was calculated by

3 $$C= {\varepsilon }_{0}{\varepsilon }_{r} \frac{A}{d} ,$$

where ${\varepsilon }_{0}$ is the permittivity of free space (or air), ${\varepsilon }_{r}$ is the relative permittivity of the substrate, A is the area of the split conducting strip, and d is the distance between the patch and the ground.

### Implementation of the law of conservation of energy

The complex Poynting vector^30^ was considered for the applied EM waves on the proposed metamaterial absorber. The average power flow was calculated by4where, ${H}_{m}$ is the maximum value of magnetic field and ŋ $\left[\approx \sqrt{\frac{\mu }{\varepsilon }}(1+j\frac{\sigma }{2\omega \varepsilon })\right]$ is the intrinsic impedance of the substrate.

To conserve energy, the real part of permittivity has to be negative, and the imaginary part has to be positive^31^, which were found accordingly. Thus (EM) energy was conserved in the absorber.

### Absorber measurement methodology

The size of the array was chosen, similar to the size of the aperture of the coaxial-to-waveguide adapter (shown in Fig. 10), to ensure effective measurement and avoid unwanted coupling^32^. Before starting measurement, the coaxial cables connected with the vector network analyzer (VNA) [Model: Agilent Network Analyzer N5227A] were calibrated with an electronic calibrator [Model: Agilent N4694-6001] using the e-cal method on the VNA. The number of points was set precisely similar to the frequency samples found from the CST simulation (1001 points) in the VNA. The S_11_ values were recorded in dB, real parts, and imaginary parts on the VNA and finally calculated using the same template found (and copied to the Excel file) from the CST simulation. All the results are plotted using Origin 9 pro software.

## Data Availability

The datasets generated and/or analyzed during the current study are not publicly available due to complexity in data understanding but are available from the corresponding author on reasonable request.
